# Access to continuous professional development for capacity building among nurses and midwives providing emergency obstetric and neonatal care in Rwanda

**DOI:** 10.1186/s12913-023-10440-8

**Published:** 2024-03-29

**Authors:** Mathias Gakwerere, Jean Pierre Ndayisenga, Anaclet Ngabonzima, Thiery Claudien Uhawenimana, Assumpta Yamuragiye, Florien Harindimana, Bernard Ngabo Rwabufigiri

**Affiliations:** 1 Regional Office for East and Southern Africa, United Nations Population Fund, 09 Simba Road, Sunninghill, Johannesburg, South Africa; 2 School of Nursing and Midwifery, College of Medicine and Health Science, University of Rwanda, Kigali, Rwanda; 3 Arthur Labatt Family School of Nursing, Western University, London, Canada; 4 JSI Research & Training Institute, Inc, International Division, Washington, DC, USA; 5 School of Health Sciences, University of Rwanda, Kigali, Rwanda; 6 United Nations Population Fund, KG 7 Ave, Kigali, Rwanda; 7 College of Medicine and Health Sciences, School of Public Health, University of Rwanda, Kigali, Rwanda

**Keywords:** CPD, EmONC, Midwifery

## Abstract

**Background:**

Nurses and midwives are at the forefront of the provision of Emergency Obstetric and Neonatal Care (EmONC) and Continuous Professional Development (CPD) is crucial to provide them with competencies they need to provide quality services. This research aimed to assess uptake and accessibility of midwives and nurses to CPD and determine their knowledge and skills gaps in key competencies of EmONC to inform the CPD programming.

**Methods:**

The study applied a quantitative, cross-sectional, and descriptive research methodology. Using a random selection, forty (40) health facilities (HFs) were selected out of 445 HFs that performed at least 20 deliveries per month from July 1st, 2020 to June 30th, 2021 in Rwanda. Questionnaires were used to collect data on updates of CPD, knowledge on EmONC and delivery methods to accessCPD. Data was analyzed using IBM SPSS statistics 27 software.

**Results:**

Nurses and midwives are required by the Rwandan midwifery regulatory body to complete at least 60 CPD credits before license renewal. However, the study findings revealed that most health care providers (HCPs) have not been trained on EmONC after graduation from their formal education. Results indicated that HCPs who had acquired less than 60 CPD credits related to EmONC training were 79.9% overall, 56.3% in hospitals, 82.2% at health centres and 100% at the health post levels. This resulted in skills and knowledge gaps in management of Pre/Eclampsia, Postpartum Hemorrhage and essential newborn care. The most common method to access CPD credits included workshops (43.6%) and online training (34.5%). Majority of HCPs noted that it was difficult to achieve the required CPD credits (57.0%).

**Conclusion:**

The findings from this study revealed a low uptake of critical EmONC training by nurses and midwives in the form of CPD. The study suggests a need to integrate EmONC into the health workforce capacity building plan at all levels and to make such training systematic and available in multiple and easily accessible formats.

**Implication on nursing and midwifery policy:**

Findings will inform the revision of policies and strategies to improve CPD towards accelerating capacity for the reduction of preventable maternal and perinatal deaths as well as reducing maternal disabilities in Rwanda.

**Supplementary Information:**

The online version contains supplementary material available at 10.1186/s12913-023-10440-8.

## Background

Continuous professional development (CPD) for capacity building is critical for health workers for enabling them to continually update their competencies to ensure they are able to meet client needs and adapt to constant changes in the practice environment [1]. CPD has been defined as any kind of education that professionals receive after completing their basic education of professional entry [2]. It is highly recommended that professionals in practice keep regularly updated about new protocols and refresh their knowledge for better continuity of care [3]. In emergency obstetric and neonatal care (EmONC), CPD is necessary to allow health care providers (HCPs) to feel confident and ready to deliver quality maternal and newborn health services [4]. Nurses and midwives, as essential providers of EmONC services, need to update their knowledge to enhance the quality of EmONC service delivery. EmONC encompasses all care provided to address emergent complications that occur during pregnancy, labor, and childbirth [4].

Globally, the maternal mortality ratio (MMR) is still unacceptably high; on average every day approximately 810 women lose their lives while giving birth [5]. Most of these deaths could be prevented if quality EmONC is provided by skilled health care professionals [5, 6]. The leading causes of maternal deaths include hemorrhage, hypertensive disorders, especially pre-eclampsia/eclampsia, sepsis, embolism and complications of unsafe abortions. Sub-Saharan Africa remains the region with the highest burden of maternal mortality and morbidities with an MMR of 542 per 100,000 live births, which is higher than the ratio of 216 per 100,000 live births globally [5]. According to the Sustainable Development Goal (SDG) 3.1, every country is expected to reduce the MMR to less than 70 per 100,000 live births and no country should have more than 140 maternal deaths per 100,000 live births by 2030 [7, 8]. To achieve this target, evidence-based high impact interventions should be implemented at scale and with the highest quality. The WHO has developed policies and guidelines for antenatal, intrapartum, and postpartum care, but the implementation of these guidelines and policies could be difficult if HCPs are not updated through CPDs [9].

In Rwanda, several health system-wide interventions such as community-based health programs, performance-based financing, community health insurance, and mentorship initiatives have been implemented to reduce the Maternal Mortality Ratio (MMR) [6]. Currently, the MMR in Rwanda is 203 per 100,000 live births [10]. However, despite the progressive improvement in reducing the maternal mortality ratio, there is still a need to triple efforts if the country is to achieve the SDG goal 3 target 1. The country is putting increased effort into training health care professionals in EmONC. Continuous Professional Development was formally introduced in 2013 with the adoption of the National CPD policy and since then EmONC has been one of main focus areas. Health Professional bodies were mandated to assess, validate and regulate the provision of CPD across the countries and every health provider is required to earn 60 CPD credits annually before renewing his/her professional license. This strategy enabled the Ministry of Health to keep health professionals’ knowledge and skills up to date hence improving the quality of maternal health services [11]. However, these CPD trainings were provided randomly without prior assessment of training needs of nurses and midwives in EmONC and maternity care.

Accordingly, the aim of this study is to assess uptake, accessibility to CPDs and knowledge and skills gaps on EmONC among midwives and nurses in Rwanda. Specifically, this study assessed the basic knowledge of these health cadres in EmONC, the areas in which nurses feel confident, how often nurses and midwives receive training, and finally, the ways they prefer to get the CPDs to renew their license to practice. Furthermore, since the country is progressing toward using technologies across the health system, the study assessed the familiarity of midwives and nurses to use Information Technology (IT) for learning purposes. The findings could provide evidence on the status of uptake of CPDs by nurses and midwives, describe challenges and best practices to inform revision of strategies to further improve CPD programs for midwives and nurses in Rwanda. In addition, the results could help to explore and deploy cost-effective training delivery models for nurses and midwives in Rwanda and in other similar contexts.

## Methods

### Study setting

Rwanda’s health system has been built on the administrative scheme with referral, provincial, district, and sub-district health facilities (public and private) and, overall, has 1,695 health facilities [10]. Forty (40) of the 444 health facilities that conducted at least 20 deliveries per month from July 1st, 2020 to June 30th, 2021 nationwide were randomly selected for the study.

### Study design

A cross-sectional study design was used to collect data at the selected health facilities. The study was conducted across selected facilities nationwide, selection was done randomly from a list of health facilities arranged per region and district to ensure equitable representation.

### Study population

Within the randomly selected health centers, up to four HCPs (midwives and nurses) who are assigned in maternity participated in the study. In total, 93% of the targeted HCPs from the 40 Health Facilities were recruited and participated in this study. Selected health posts had only one nurse providing health care services instead of four planned for the survey. This research excluded all participants with experience less than six months at a given health facility as they may not yet have been familiar with the facility systems and various training opportunities for EmONC.

### Sample size and sampling strategy

A random sampling method was used to enroll 40 health facilities in the study. After getting permission/authorization from District Health Offices, the recruitment was done through directors/gatekeepers of selected health facilities. The support letter from Ministry of Health was secured and was presented at each health facility chosen. Using this support letter, the researchers approached selected health facilities’ leaders (gatekeepers), including medical directors, directors of nursing and head of health centers. They asked them to assist in identifying the potential and eligible participants. Those gatekeepers did the first contact with potential participants. Data collectors ensured that each participant meet the eligibility criteria before enrolling him/her in the study to avoid selection biases.

### Data collection process

The data collection tool was piloted/tested in one health facility one week prior to data collection. Two nurses and two midwives were selected to respond to the questionnaire for testing and validation purposes. This informed the revision and finalization of the questionnaire by the research team.

The research team deployed two enumerators per each selected health facility to collect data using the validated questionnaire. Data collection began by an introduction of enumerators to potential respondents per the head of the health facility. Those potential respondents were provided with information about the research study including its objectives and they were given an official consent form requesting him/her to participate in this study if s/he agrees. After consenting, a questionnaire was administered to collect quantitative data related to the study objectives. The electronic questionnaire was developed and uploaded in the KoBo toolbox. KoBo toolbox is an open-source tool for collecting data using mobile phones. The questions were accessed via a web application link. The collected data were uploaded onto a password-protected Data were collected from 22 November 2021 to 26 November 2021.

### Data Analysis

Data analysis was preceded by data quality assurance which was done regularly throughout the data collection period. This was enabled by the KoBo toolbox which would push real time data into a national server. Study questions and related data were organized, collected and analyzed according to study objectives. Data set was accessed via a web application link. The collected data were stored onto a password-protected server for confidentiality and ethical purposes. Data were analyzed using IBM SPSS statistics 27 software. The analysis generated standard descriptive and frequencies on the key assessment indicators on uptake of CPDs, accessibility to CPDs and knowledge and skills gaps on EmONC among nurses and midwives. Data are presented using tables and graphs.

### Ethical consideration

Ethical clearances from the Rwanda National Ethical Committee was granted to researchers to conduct this study (Approval Notice: No. 984/RNEC/2021). In addition, an authorization of the health facility to collect data was secured prior to conducting interviews. Data collectors adhered to principles of confidentiality and ethics in data collection. No person’s name (except for the identification of the data collector) was recorded on any of the interviews. Permission to enter each facility, interview the different employees, and review registers was requested from the director or staff in charge of the health facility at the beginning of each visit. The data collection teams carried with them official letters of cooperation from the Ministry of Health and district level offices. Interviewees were requested to read an information note and sign a consent form before proceeding with the interview. No incentives were provided to participants hence the participation was voluntary. Respondents were able to withdraw their participation anytime during the interview. The information notes, and consent forms are added as annexes for easy reference.

## Results

### Demographic characteristics of the respondents

The study was carried out across 40 health facilities and 149 health care providers (HCP) participated in the study (Annex 1). Of them, 99 (64.4%) were female and 50 (33.6%) were male. The highest proportion of the HCP who participated in the study were less than 30 years of age, 38 (25.5%). The majority of the participants were nurses, 104 (69.8%), while the remaining 45 (30.2%) were midwives. Among the nurses, 21 (20.2%) had a high school diploma, 74 (71.1%) had an advanced diploma, and 9 (8.7%) had a bachelor’s degree. Among midwives, 42 (93.3%) of them had advanced diplomas and 3 (6.7%) had bachelor’s degrees in midwifery. The highest proportion of the HCP who participated in the study had been working in maternity services for more than 5 years and were 96 (64.4%). The detailed results are shown in Table 1 below.

**Table 1:** Socio-demographic characteristics

**Sex of the respondents [n (%)]**	
Female	99 (64.4%)
Male	50 (33.6%)
**Age group [n (%)]**	
Less 30 years	38 (25.5%)
30–34 years	25 (16.8%)
34–39 years	31 (20.8%)
40–44 years	29 (19.5%)
45–49 years	15 (10.1%)
50 + years	11 (7.4%)
**Professional qualification [n (%)]**	
Nurse	104 (69.8%)
Midwife	45 (30.2%)
**Educational level [n (%)]**	
Nurse (n = 104)	
High school diploma (A2)	21 (20.2%)
Advanced diploma (A1)	74 (71.1%)
Bachelor’s degree (A0)	9 (8.7%)
Midwife (n = 45)	
Advanced diploma (A1)	42 (93.3%)
Bachelor’s degree (A0)	3 (6.7%)
**Years of experience in maternity services [n (%)]**	
1 year or less	17 (11.4%)
Between 1 and 3 years	29 (19.5%)
Between 3 and 5 years	7 (4.7%)
More than 5 years	96 (64.4%)
**Type of health facility**	
DH	4 (10%)
HC	35 (87.6%0
HP	1 (2.4%)

### Respondents’ obstetric experience and skills in EmONC

Overall, the predominant number of births that the HCPs conduct themselves per week was between one and five (71.1%). Broken down by the type of health facility, a large number of the respondents said that they perform between 5 and 10 (50%) for the hospitals, between one and five (74.4%) for the health centers and between one and five (75.0%) for the health posts. The majority of the HCPs felt confident in performing a birth-related diagnosis on their own (89.9%): all the HCPs from the hospitals felt confident, while 91.5% felt confident at the health centers and none felt confident at the health posts. Similarly, those who felt confident in performing births on their own was 89.9% overall, with all of the HCP from the hospitals feeling confident, and 89.9% from the health centers and 25% from the health posts. Across all the types of facility, the majority of the HCPs agreed that birth complications while performing deliveries sometimes happen, specifically as follows: overall (90.6%), hospital (93.8%), health center (90.7%) and health post (75.0%). In terms of diagnostic and management, the majority of the HCP agreed that they feel less confident with pre-eclampsia, with 67.1% overall, 31.3% at the hospitals, 71.3% at the health centers and 75.0% at health posts. Among the newborn health concerns, the majority of the HCP were less confident in immediate care after delivery for baby who does not cry, mainly 74.5% overall, 37.5% in hospitals, 78.3% in health centers and 100% in health posts were less confident on immediate care after delivery for baby who does not cry. Table 2 shows the results in details.

**Table 2:** Respondents’ obstetric experience and skills in EmONC

	District hospital n (%)	Health center n (%)	Health post n (%)	Total n (%)
**Average number of births conducted per week**				
Less than 1	0 (0.0%)	5 (3.9%)	0 (0.0%)	5 (3.4%)
Between 1 and 5	7 (43.8%)	96 (74.4%)	3 (75.0%)	106 (71.1%)
Between 5 and 10	8 (50%)	20 (15.5%)	0 (0.0%)	28 (18.8%)
More than 10	1 (6.2%)	8 (6.2%)	1 (25%)	10 (6.7%)
**Number of HCP who felt confident in performing a birth –related diagnosis on their own**				
Yes	16 (100%)	118 (91.5%)	0 (0.0%)	134 (89.9%)
No	0	11 (8.5%)	4 (100%)	16 (10.7%)
**Number of HCP who felt Confident in performing birth on their own**				
Yes	16 (100%)	116 (89.9%)	1 (25%)	133 (89.9%)
No	0	13 (10.1%)	3 (75%)	15 (10.1%)
**Frequency to experience birth complication while performing normal delivery**				
Never	0 (0.0%)	8 (6.2%)	1 (25.0%)	9 (6.0%)
Sometimes	15 (93.8%)	117 (90.7%)	3 (75.0%)	135 (90.6%)
Often	1 (6.3%)	4 (3.1%)	0 (0.0%)	5 (3.4%)
**Area of maternal health in which HCP feel less confident**				
Diagnostic and management of pre/eclampsia	5 (31.3%)	92 (71.3%)	3 (75%)	100 (67.1%)
Diagnostic and management of Puerperal sepsis	4 (25%)	67 (51.9%)	2 (50%)	73 (49.0%)
Diagnostic and management of Postpartum Hemorrhage	4 (25%)	73 (56.6%)	2 (50%)	79 (53%)
Diagnostic and management of obstructed labor	8 (50%)	75 (58.1%)	4 (100%)	87 (58.4%)
Other*	1 (6.3%)	2 (1.6%)	0 (0.0%)	3 (2.0%)
None	4 (25%)	9 (7.0%)	0 (0.0%)	13 (8.7%)
**Area of Newborn health in which HCP feel less confident**				
Prevention of hypothermia	1 (6.1%)	18 (14%)	4 (100%)	19 (12.8%)
Prevention of infections	0 (0.0%)	41 (31.8%)	1 (25%)	42 (28.25%)
Immediate care after delivery for baby who cried	0 (0.0%)	7 (5.4%)	0 (0.0%)	7 (4.7%)
Immediate care after delivery for baby who did not cry	6 (37.5%)	101 (78.3%)	4 (100%)	111 (74.5%)
None	9 (56.3%)	15 (11.6%)	0 (0.0%)	24 (16.1%)

* Other areas include bleeding during pregnancy, infection during pregnancy, Manual Vacuum Aspiration, Cord prolapse, Management of labor in case of fetal distress, Post abortion care and Use of Cardiotocography TG and use of Echography

### Respondents’ previous training on EmONC related topics

Overall, the majority of the HCPs reported having never being trained on EmONC after graduation from their formal education. Apart from the hospitals, where most of the HCP were trained, in the two years (37.5%) and within 2 and 5 years (37.5%), at health centers and health posts, the majority of the HCP had never been trained on EmONC after graduation from their formal education. Among the relevant EmONC competencies, the overall majority of the HCP had been trained on B EmONC while 28.8% had not been trained on any of the topics. The proportion of HCP who were not trained on any topics is 12.5% for the hospitals, 30.2% for the health centers and 50% for the health posts. Of all the HCP, 63.1% had benefited from a mentorship program on EmONC after graduation. The Table 3 below details the findings.

**Table 3:** Respondents’ training experience

	District hospital n (%)	Health center n (%)	Health post n (%)	Total n (%)
**Number of HCP trained on BEMONC per category of HFs**				
Less 25%	4 (25%)	29 (22.5%)	4 (25%)	4 (25%)
25-50%	5 (31.3%)	50 (38.8%)	5 (31.3%)	5 (31.3%)
50-75%	7 (43.8%)	13 (10.1%)	7 (43.8%)	7 (43.8%)
Above75%	0 (0.0)	37 (28.7%)	0 (0.0)	0 (0.0)
**1. Time elapsed since HCP participated in a training on EmONC**				
In the 2 last year	6 (37.5%)	44 (34.1%)	0 (0.0)	50 (33.6%)
Within 2 and 5 years ago	6 (37.5%)	16 (12.4%)	1 (25%)	23 (15.4%)
More than 5 years ago	0 (0.0)	20 (15.5%)	1 (25%)	21 (14.1%)
Never after graduation	4 (25%)	49 (38.0%)	2 (50%)	55 (36.9%)
**Number of HCP trained/oriented in the maternal health life saving**				
B-EmONC/HMS	10 (62.5%)	62 (48.1%)	2 (50%)	74 (49.7%)
ENC/HBB/HBS	14 (87.5%)	57 (44.2%)	1 (25%)	72 (48.3%)
ANC/FANC	6 (37.5%)	47 (36.4%)	1 (25%)	54 (36.2%)
PAC/MVA	9 (56.3%)	40 (31.0%)	2 (50%)	51 (34.2%)
Not trained	2 (12.5%)	39 (30.2%)	2 (50%)	43 (28.8%)
PNC	5 (31.3%)	35 (27.1%)	1 (25%)	41 (27.5%)
**Number of HCP who benefited from a mentorship program on EmONC after graduation**				
Yes	12 (75%)	80 (62%)	2 (50%)	94 (63.1%)
No	4 (25%)	49 (38%)	2 (50%)	55 36.9%)

### Ways respondents accumulate CPD credits

Normally, every nurse or midwife is required to complete at least 60 credits to be able to renew his / her license. This study revealed that the HCP who had acquired less than 60 CPD credits related to EmONC training were the most predominant, with 79.9% overall, 56.3% at the hospitals, 82.2% at the health centers and 100% at the health posts. The most common way of delivering the CPD credits was through workshops (43.6%) and online training (34.5%). Most of the HCP noted that it was hard for them to achieve the required CPD credits (57.0%). The detailed results are presented in Table 4 below.

**Table 4:** Ways respondents accumulate CPD credits

	District hospital n (%)	Health center n (%)	Health post n (%)	Total n (%)
**Number of HCP who acquired CPD credits related to EmONC training (Considering the last 5 years for those who have been serving for longer) after graduation**				
Less 60 credits	9 (56.3%)	106 (82.2%)	4 (100%)	119 (79.9%)
60%-100 credits	3 (18.7%)	12 (9.3%)	0 (0.0%)	15 (10.1%)
Above 100 credits	4 (25.0%)	11 (8.5%)	0 (0.0%)	15 10.1%)
**How have these CDP credits been delivered?/ Methodology used in delivering the attended CDP sessions**				
Workshop	13 (81.3%)	50 (38.8%)	2 (50%)	65 (43.6%)
Online Training	9 (56.3%)	42 (36.6%)	1 (25%)	52 (34.9%)
Conference	0 (0.0%)	11 (8.5%)	0 (100%)	11 (7.4%)
All of the above	0 (0.0%)	7 (5.4%)	0 (100%)	7 (4.7%)
None of the above	2 (12.5%)	41 (31.8%)	1(25%)	44 (29.5%)
Staff meeting	0 (0.0%)	3 (2.3%)	2 (50%)	5 (3.4%)
**Level of difficulty in accumulating CPD credits**				
Very easy	3 (18.8%)	8 (6.2%)	1(25%)	12 (8.1%)
Quite easy	7 (43.8%)	30 (23.3%)	0(0.0%)	37 (24.8%)
Hard	6 (37.5%)	76 (58.9%)	3 (75%)	85 (57.0%)
Extremely hard	0 (0.0%)	15 (11.6%)	0 (00%)	15 (10.0%)

### Respondents’ digital literacy and access to digital devices

The most common electronic device owned by the HCP was a smartphone (98.7%). Overall, there were no significant differences between the places where the HCP usually accessed the internet. However, accessing the internet at work was most frequent for the hospitals (43.7%) and health centers (46.5%), and least frequent for the health posts (0.0%). The majority of the HCP, agreed to be comfortable using a computer (57.0%), a normal feature phone (83.2%) and smartphone (86.6%) as tools that facilitate eLearning. Similarly, being comfortable with eLearning was predominant overall (51.0%), at the hospitals (75.0%) and health centers (48.8%), however majority of the heath posts HCP agreed to be somewhat comfortable with eLearning (50.0%). Furthermore, the HCP aged less than 40 years are the ones being more comfortable with Electronic-Learning. The details are provided below in Table 5.

**Table 5:** Respondents’ digital literacy and access to digital devices

	District hospital n (%)	Health center n (%)	Health post n (%)	Total n (%)
**Types of electronic devices owned by respondents**				
Smartphone	14 (87.5%)	129 (100%)	4 (100%)	147 (98.7%)
Feature phone	6 (37.5%)	49 (38.0%)	0 (0.0%)	55 (36.9%)
Computer	3 (18.7%)	27 (20.9%)	0 (0.0%)	30 (20.1%)
Tablet	0 (0.0%)	16 (12.4%)	0 (0.0%)	16 (10.7%)
**Places where respondents usually access internet**				
At work	7 (43.7%)	60 (46.5%)	0 (0.0%)	67 (45.0%)
Both home and work	4 (25%)	54 (41.9%)	2 (50%)	60 (40.3%)
Internet cafe	6 (37.5%)	51 (39.5%)	2 (50%)	59 (39.6%)
**Respondents’ comfortability in using a computer**				
Yes	9 (56.2%)	74 (57.4%)	2 (50%)	85 (57%)
No	1 (6.3%)	13 (10.1%)	1 (25%)	15 (10.1%)
Somehow comfortable	6 (37.5%)	42 (32.5%)	1 (25%)	49 (32.9%)
**Respondents’ comfortability in using a normal feature phone**				
Yes	12 (75%)	111 (86%)	1 (25%)	125 (83.2%)
No	2 (12.5%)	9 (7%)	0 (0.0%)	11 (7.4%)
Somehow comfortable	2 (12.5%)	9 (7%)	3 (75%)	14 (9.4%)
**Respondents’ comfortability in using a smartphone**				
Yes	15 (93.8%)	111 (86%)	3 (75%)	129 (86.6%)
No	0 (0.0%)	3 (2.3%)	0 (0.0%)	3 (2.0%)
Somehow comfortable	1 (6.3%)	15 (11.6%)	1 (25%)	17 (11.4%)
**Respondents’ comfortability in E- learning**				
Yes	12 (75%)	63 (48.8%)	1(25%)	76 (51.0%)
No	2 (12.5%)	38 (29.5%)	1 (25%)	41 (27.5%)
Somehow comfortable	2 (12.5%)	28 (21.7%)	2 (50%)	32 (21.5%)
**Respondents’ comfortability in E-learning per age group**				
Less 30 years	3 (25.5%)	23 (36.5%)	1 (100%)	27 (35.5%)
30–34 years	4 (33.3%)	13 (20.6%)	0 (0.0%)	17 (22.4%)
34–39 years	5 (41.7%)	11 (17.5%)	0 (0.0%)	16 (21.1%)
40–44 years	0 (0.0%)	8 (12.7%)	0 (0.0%)	8 (10.5%)
45–49 years	0 (0.0%)	6 (9.5%)	0 (0.0%)	6 (7.9%)
50 + years	0 (0.0%)	2 (3.5%)	0 (0.0%)	1 (2.6%)

### Uptake of E-learning

Of all the HCP, 72.5% use their computer or smartphone to access eLearning. Of these, a proportion (23.1%) dedicated more than 30 min per day to self-training in EmONC using their phone. However, at the hospitals, a larger proportion would dedicate a few minutes in a week (30.0%) and more than an hour in a week (30.0%). Overall, more HCP had not used eLearning as a teaching-learning method in the past two years (37.3%). However, those who had never used it are more at the health centers (39.5%) and health posts (75.0%) but the eLearning utilization is high at the hospitals with 43.8% who have used it more than four times in the past two years. Supplemental knowledge/skills in existing areas was the predominant (80.5%) expectation from the phone delivered eLearning EmONC modules, while learning new learning and teaching methods was the least expectation (51.0%). At the health centers, 55.8% of the HCP would be able and willing to self-support for their personal remote training. Inconsistently, 43.8% at the hospitals as well as 100% at the health posts would not be able and willing to self-support for their personal remote training. Table 1 in the annex shows the findings in detail.

### Respondents’ basic knowledge on EmONC

While assessing the basic information on EmONC, specifically by assessing what aspect should be given special attention during abdominal examination of an at-term pregnant mother, the majority of the HCP agreed that all elements (fundal height, descent of the presenting part, fetal heart tones and frequency and duration of contractions) should be given special attention. However, at the health centers and health posts, there were some HCP who believe that not all of those elements should be given special attention (for example: 17.1% of the HCP from health centers agreed that only fundal height should be given special attention). In addition, the majority of the HCP defined postpartum hemorrhage as vaginal bleeding of more than 500 mL after vaginal birth, 95.3% overall, 100% at the hospitals, 84.6% at health centers and 100% at the health posts. Table 2 in the annex summarizes the findings.

## Discussion

The purpose of the study was to assess the progress of uptake and accessibility of midwives and nurses to CPD and determine their knowledge and skills gaps in key competencies of EmONC in Rwanda. Generally speaking, the study findings revealed a low to moderate progress of uptake of CPDs among nurses and midwives’ health cadres due to lack of opportunity or difficult access. The study also revealed knowledge and skills gaps in critical competencies for EmONC; which calls for a particular focus on EmONC during CPD programming. The study findings indicated that the majority of nurses have never received any formal training on EmONC after graduation from their formal education. The proportion of HCP who were not trained on any topics is 12.5% for the hospitals, 30.2% for the health centers and 50% for the health posts. This calls for more investments in CPDs opportunities tailored to the needs of individual health care providers practicing EmONC in a given health facility. Whichever delivery method is used, CPD are important to keep practitioners updated and enhance their practice [12]. According to Gray et al. [1] adult learners need a structured teacher guided approach compared to the youngest clinicians who can comply with different modes of teaching, including self-directed learning. Similarly, the study revealed small progress of uptake of mentorship on EmONC with only 63.1% health providers mentored on EmONC after graduation. Small coverage of mentorship is another important missed opportunity because mentorship proved to be a cost effective approach to transfer skills between the most experienced professional to less experienced one in a more sustainable manner [13–15]. This might have been due to various factors including lack of systematic plan for mentorship scale up and financial constraints .

As such, this form of training could complement the continuous professional development delivered through traditional training. Similar strategies have been implemented in other countries with similar contexts. For example, a cascade type mentorship for health workers has been implemented in Uganda and demonstrated tremendous results in terms of maternal and neonatal health outcomes. Nevertheless, given the nature and complex set of practical skills required for better management of obstetric complications, clinical mentorship for EmONC should remain erratic, dynamic and always adapted to the case in hand and to the competencies and skills gaps of the health care provider [13, 16, 17].

Despite the fact that Pre/Eclampsia and Postpartum hemorrhage remain among the main killers of women during delivery at global level and in Rwanda, this study revealed knowledge and skills gaps in management of both critical obstetric conditions among nurses and midwives. Nurses and midwives reported feeling less confident in some aspects of EmONC, including management of eclampsia, and care of newborn after birth especially when babies don’t cry immediately [5]. Organizing CPDs trainings in management of these life threatening conditions could probably make health providers feel confident to provide effective quality EmONC services thus contribute to acceleration of reduction of preventable maternal and neonatal deaths in Rwanda towards achieving the SDG goals and targets [18–20].

These findings correlate also with another study conducted in Australia where participants suggested more training in the management of eclampsia [21]. Moreover, the skill gaps in management of key obstetric complications could be explained by a mismatch between the CPDs and the needs on the field. CPDs opportunities are limited in Rwanda and they are not always aligned to the felt need of nurses and midwives on ground. Similar mismatches between CPD needs and supply has been reported by Feldacker et al. [22] in a study conducted in Malawi, Tanzania and South Africa due to lack of evidence-based planning and effective coordination. The Ministry of Health of Rwanda has put in place incentives to motivate health care providers to invest in lifelong learning. One of them is an instruction to earn at least 60 CPD credits before renewing the license to practice nursing and midwifery in Rwanda [23]. However, more than 79.9% face the challenges of having necessary credits when planning to renew their license to practice, depending on the working place across the health system. The study findings indicate that among respondents who reported facing challenges to earn the required minimum of CPDs credits 56.3% were from the hospitals, 82.2% from the health centers and 100% from the health posts. The most common way of delivering the CPD credits was through workshops (43.6%) and online training (34.5%). Most of the HCPs noted that it was hard for them to achieve the required CPD credits (57.0%), suggesting a need for an organizational culture that supports CPDs. For participants who received training, common methodologies were face-to-face and online teaching. Regarding the common devices participants use for online training, the majority use their smartphones, while others use a computer. Since internet connectivity is a challenge, respondents in the study mentioned using those devices mostly at the working places to benefit internet connectivity at work [24]. Similarly, Addae et al. [25] reported in a study conducted in Ghana on online learning experience for nurses and midwives’ students that the lack of internet connectivity was one of the major challenges that impeded the programme. These findings highlight untapped potential of using advanced information technological systems to deliver CPDs courses in a more cost-effective manner as virtual training represents only 34.5% due to small coverage and cost of using the internet by the health worker’s community. This calls for more investment in IT infrastructures such as optic fibers and other cost-effective means of expanding internet coverage across the country. In the meanwhile, tools such as safe delivery app [26] that provide nurses and midwives evidence-based and up-to-date clinical guidelines could be used as these don’t require internet when successfully downloaded. Findings from this study correlate with a study on the use of blended learning in nursing where poor IT skills and lack of organizational support were identified as weakness to CPD provision among nurses and midwives [27–29].

The study findings call for more investment in enhancement of skills and capacities of human resource for health using more predictable and sustainable approaches such as CPDs and mentorship. This will require integration of these interventions in health sector policies and strategies, allocation of finances and strong monitoring and evaluation of the impact of these interventions on health service delivery and quality of care. Moreover, the study revealed challenges in the use of e-learning as a tool to sustain CPDs due to the limited internet connectivity. No one would deny the potential of technology in health care delivery and more lessons and best practices have been gathered during the COVID-19 pandemic era. This calls for the Government of Rwanda to continue strengthening ICT infrastructure and deploy strategies to ease the finance burden for the user.

### Strength and limitations

This study has been conducted using a random sampling from all facilities with significant obstetric activity across the country arranged per region and district to ensure equitable representation.

This enabled researchers to collect information from reliable sources while minimizing biases hence with increased validity and generalizability. However, since the study relied on self-assessment/reporting of knowledge and skills, the research team acknowledged some of the limitations of the study including respondent bias as participants might have reported what he/she felt could meet the expectations of the researchers. In addition, due to financial constraints, the study sample covered 40 out of 444 health facilities which could have affected the generalizability of the findings. Besides the general survey related limitation, evidence suggests that increased knowledge for healthcare professionals does not automatically translate into improvements in clinical practice or patient outcomes. Nevertheless, literature supports CPD in terms of accumulation and how clinicians apply the knowledge gained in practice [30]. Therefore, there is a need for more studies to explore and evaluate how the CPD straining translates in actual change in practices and its overall impact on reduction of preventable maternal and neonatal deaths in Rwanda.

## Conclusion

The main purpose of this study was to assess the CPD program for nurses and midwives in the context of EmONC in Rwanda. The findings highlight a modest progress of the program due to lack of systematic policy and strategic approach for its implementation. This resulted in skills and knowledge gaps in management of 3 critical and life threatening obstetric conditions namely Pre/Eclampsia, PPH, essential newborn care. The study revealed that Nurses and midwives face challenges to accumulate the number of credits required to renew their licenses to practice due to limited opportunities for CPDs. Online teaching was identified as an alternative methodology for CPD training delivery. However, participants mentioned internet access as a barrier to effective online learning. Therefore, the study findings are a call for policy makers to integrate CPDs for nurses and midwives in policies and strategies and allocate enough resources to ensure systematic implementation. These will require a multisector approach to ensure CPDs are prioritized and financed as well as an infrastructure system is put in place to facilitate uptake of CPDs in a cost-effective manner.

### Electronic supplementary material

Below is the link to the electronic supplementary material.

Supplementary Material 1

Supplementary Material 2

## Data Availability

The datasets used and/or analyzed during the current study available from the corresponding author on reasonable request.
