# Supplementary material for: Access to continuous professional development for capacity building among nurses and midwives providing emergency obstetric and neonatal care in Rwanda
Source: BMC Health Serv Res. 2024 Mar 29;24:394. doi: 10.1186/s12913-023-10440-8 (PMC10979581; doi:10.1186/s12913-023-10440-8)
Supplement: Supplementary file 1 — Supplementary Material 1 [file 12913_2023_10440_MOESM1_ESM.docx]

*Appendices 1. Table 1. Respondents' E-learning capability*

|  | District hospital  n (%) | Health center  n (%) | Health post  n (%) | Total  n (%) |
| --- | --- | --- | --- | --- |
| 1. Utilization of personal computer or smartphone to E-learning | | | | |
| Yes | 10 (62.5%) | 95 (73.6 %) | 3 (75 %) | 108 (72.5%) |
| No | 6 (37.5%) | 34 (26.4%) | 1 (25%) | 41 (27.5%) |
| 1. If response to the above question is YES, How much time would you dedicate to self-training in EmONC using your phone. | | | | |
| None | 0 (0.0%) | 6 (6.3%) | 2 (66.7%) | 8 (7.4%) |
| 5-10 mins per day | 1 (10%) | 12 (12.6%) | 0 (0.0%) | 13 (12%) |
| 10-20 mins per day | 1 (10%) | 7 (7.4%) | 0 (0.0%) | 8 (7.4%) |
| 20-30 mins per day | 0 (0.0%) | 23 (24.2%) | 0 (0.0%) | 23 (21.3%) |
| More than 30 mins per day | 2 (20%) | 22 (23.2%) | 1 (33.3%) | 25 (23.1%) |
| Few minutes in a week | 3 (30%) | 21 (22.1%) | 0 (0.0%) | 24 (22.2%) |
| More than an hour in a week | 3 (30%) | 4 (4.2%) | 0 (0.0%) | 7 (6.5%) |
| 1. Frequency of using E-Learning as a teaching-learning method in the past 2 years | | | | |
| Never | 3 (18.8%) | 51 (39.5%) | 3 (75%) | 57 (37.3%) |
| 1-4 times | 6 (37.5%) | 42 (32.6%) | 0 (0.0%) | 48 (32.2%) |
| 5+ times | 7 (43.8%) | 36 (27.9%) | 1 (25%) | 44 (29.5%) |
| 1. Respondents' expectations from the phone-delivered e-learning EmONC modules | | | | |
| Supplement knowledge/skills in existing knowledge area | 16 (100 %) | 102 (79.1 %) | 2 (50%) | 120(80.5%) |
| Acquire knowledge/skills in a new knowledge area | 16 (100 %) | 84 (65.1 %) | 1 (25%) | 101 (67.8%) |
| Fulfill course requirement to earn CPD credits | 16 (100 %) | 80 (62 %) | 1 (25%) | 97 (65.1%) |
| Learn new learning/teaching method | 15 (93.8 %) | 61 (47.3 %) | 0 (0.0%) | 76 (51%) |
| 1. Ability and willingness to self- support for respondents' personal remote training | | | | |
| No | 7 (43.8 %) | 28 (21.7%) | 4 (100%) | 39 (26.2%) |
| Yes, but only below 500 Rwf per module | 4 (25%) | 72(55.8%) | 0 (0.0%) | 76 (51%) |
| Yes, between 500 Rwf per 2,000 Rwf per module | 4 (25 %) | 18 (14.0%) | 0 (0.0%) | 22 (14.8%) |
| Yes, more than 2,000 Rwf | 1(6.3%) | 11 (8.5%) | 0 (0.0%) | 12 (8.1%) |

*Appendices 2 Table 2. Basic information on EmONC*

|  | District hospital  n (%) | Health center  n (%) | Health post  n (%) | Total  n (%) |
| --- | --- | --- | --- | --- |
| 1. During abdominal examination of term pregnant mother, special attention should be given to | | | | |
| Fundal height, which will helps confirm gestational age or indicate size-date discrepancy | 0 (0.0%) | 22 (17.1 %) | 0 (0.0%) | 22 (14.8%) |
| Descent of the presenting part, which would help in evaluating progress of labor | 0 (0.0%) | 2 (1.6 %) | 0 (0.0%) | 2 (13%) |
| Fetal heart tones, which will help indicate fetal condition | 0 (0.0%) | 13 (10.1 %) | 1 (25 %) | 15 (10.1%) |
| Frequency and duration of contractions to determine quality of contractions and help determine stage/phase of labor, as well as evaluate progress of labor | 0 (0.0%) | 5 (3.9 %) | 0 (0.0%) | 5 (3.4%) |
| All the above | 16 (100 %) | 87 (67.4%) | 3 (75 %) | 1. (70.5%) |
| 1. Postpartum hemorrhage is defined as | | | | |
| vaginal bleeding of any amount after childbirth | 0 (0.0%) | 1 (0.8%) | 0 (0.0%) | 1(0.7%) |
| sudden bleeding after childbirth | 0 (0.0%) | 2 (1.6%) | 0 (0.0%) | 2 (1.4%) |
| vaginal bleeding in excess of 300 mL after childbirth | 0 (0.0%) | 4 (3.2%) | 0 (0.0%) | 4 (2.7%) |
| Vaginal bleeding in excess of 500 mL after vaginal birth | 16 (100 %) | 129 (94.6%) | 4 (100.0%) | 142 (95.3%) |
