# Supplementary material for: Access to continuous professional development for capacity building among nurses and midwives providing emergency obstetric and neonatal care in Rwanda
Source: BMC Health Serv Res. 2024 Mar 29;24:394. doi: 10.1186/s12913-023-10440-8 (PMC10979581; doi:10.1186/s12913-023-10440-8)
Supplement: Supplementary file 2 — Supplementary Material 2 [file 12913_2023_10440_MOESM2_ESM.docx]

## APPENDICES 3: Randomly Selected facilities for quantitative data collection

| **Province** | **District** | **Sub-District** | **Health facility** | **Type** | **MNH SERVICE** | **Alternative HC** |
| --- | --- | --- | --- | --- | --- | --- |
| East | Bugesera District | Nyamata Sub District | Ntarama CS | CS | OK |  |
| East | Bugesera District | Nyamata Sub District | Ramiro HP | HP | NOT | Gashora HC |
| North | Burera District | Butaro Sub District | Rugengabali HP | HP | NOT | Rugengabali HC |
| North | Burera District | Butaro Sub District | Kamanyana HP | HP | OK |  |
| North | Gakenke District | Ruli Sub District | Gihinga HP | HP | NOT | Coko HC |
| North | Gakenke District | Ruli Sub District | Huro HP | HP | NOT | Muhondo HC |
| North | Gakenke District | Ruli Sub District | Ruli DH | DH | OK |  |
| North | Gakenke District | Nemba Sub District | Nemba DH | DH | OK |  |
| Kigali City | Gasabo District | Kibagabaga Sub District | Gatsata CS | CS | OK |  |
| Kigali City | Gasabo District | Kibagabaga Sub District | Kidashya HP | HP | NOT | Nyacyonga HC |
| Kigali City | Gasabo District | Kibagabaga Sub District | Kinyinya CS | CS | OK |  |
| East | Gatsibo District | Kiziguro Sub District | Bugarura CS | CS | OK |  |
| South | Gisagara District | Kibilizi Sub District | Mugombwa CS | CS | OK |  |
| South | Gisagara District | Kibilizi Sub District | Kibilizi (gisagara) CS | CS | OK |  |
| South | Gisagara District | Kibilizi Sub District | Kibilizi DH | DH | OK |  |
| South | Gisagara District | Gakoma Sub District | Gakoma DH | DH | OK |  |
| South | Kamonyi District | Remera Rukoma Sub District | Mugina HP | HP | NOT | Mugina HC |
| West | Karongi District | Kibuye Sub District | Manji HP | HP | NOT | Mukungu HC |
| Kigali City | Kicukiro District | Masaka Sub District | Masaka HP | HP | NOT | Busanza HC |
| East | Kirehe District | Kirehe Sub District | Mahama CS | CS | OK |  |
| South | Muhanga District | Kabgayi Sub District | Musange HP | HP | NOT | Mushishiro HC |
| South | Muhanga District | Kabgayi Sub District | Shyogwe CS | CS | OK |  |
| East | Ngoma District | Kibungo Sub District | Karembo HP | HP | NOT | Remera HC |
| West | Ngororero District | Kabaya Sub District | Hindiro CS | CS | OK |  |
| West | Nyabihu District | Shyira Sub District | Birembo CS | CS | OK |  |
| West | Nyabihu District | Shyira Sub District | Shyira CS | CS | NOT | Rwankeri HC |
| East | Nyagatare District | Nyagatare Sub District | Nyakigando (Nyagatare) CS | CS | OK |  |
| East | Nyagatare District | Nyagatare Sub District | MBALE HP | HP | NOT | Rukomo HC |
| East | Nyagatare District | Nyagatare Sub District | Nkoma HP | HP | NOT | Muhambo HC |
| South | Nyamagabe District | Kaduha Sub District | Buruhukiro CS | CS | OK |  |
| West | Nyamasheke District | Kibogora Sub District | Ruheru (kanjongo Nyamasheke) CS | CS | OK |  |
| West | Nyamasheke District | Kibogora Sub District | JARAMA HP | HP | NOT | Karengera HC |
| West | Nyamasheke District | Bushenge Sub District | SAVE HP | HP | NOT | Kamonyi HC |
| South | Nyaruguru District | Munini Sub District | Rubona (Ngoma) HP | HP | NOT | Ngoma HC |
| West | Rubavu District | Gisenyi Sub District | Busasamana CS | CS | OK |  |
| South | Ruhango District | Ruhango Sub District | Kigoma CS | CS | OK |  |
| North | Rulindo District | Kinihira Sub District | Mubuga (Rulindo) HP | HP | NOT | Tare HC |
| West | Rusizi District | Gihundwe Sub District | NYAMUZI HP | HP | NOT | Giheke HC |
| West | Rusizi District | Gihundwe Sub District | Gihundwe CS | CS | OK |  |
| West | Rutsiro District | Murunda Sub District | GITWA (MUSHUBATI) HP | HP | OK |  |
